# A rare case of Immunoglobulin A dominant post-infectious glomerulonephritis (IgA PIGN) in a young patient

**DOI:** 10.1186/s12882-022-02965-7

**Published:** 2022-10-17

**Authors:** A. Saghar, G. Klaus, B. Trutnau, M. Kömhoff, H. J. Gröne, S. Weber

**Affiliations:** 1 KfH-Renal Centre for Children and Adolescents, Marburg, Germany; 2 University Hospital Marburg, Clinic II for Paediatric Nephrology, Marburg, Germany; 3 Philipps University Marburg, Institute of Pharmacology, Marburg, Germany

**Keywords:** Proteinuria, Immunoglobulin A dominant postinfectious glomerulonephritis, Renal failure

## Abstract

**Background:**

Immunoglobulin A dominant postinfectious glomerulonephritis (IgA PIGN) is a unique medical entity that is rare in the paediatric population. It usually presents with severe renal failure, heavy proteinuria, hypertension, and hypocomplementemia and frequently has an unfavourable prognosis. IgA PIGN generally occurs in association with staphylococcal infections and diabetes mellitus in adult patients. Other pathogens include *Escherichia coli* and *Streptococcus* sp. Immunofluorescence studies of kidney biopsy samples show IgA as dominant or codominant antibody.

**Case presentation:**

We encountered a 3-year-old girl with IgA PIGN presenting with acute renal failure, oedema, hypertension, and heavy proteinuria of 7955 mg/g creatinine. Renal biopsy specimens showed diffuse glomerular endocapillary hypercellularity with prominent neutrophil and monocyte infiltration on light microscopy. Strong deposits of IgA and C_3_ were observed along the glomerular basement membranes and the mesangium by immunofluorescence microscopy, and electron microscopy revealed the presence of subepithelial humps.

The patient was managed with steroid (and probatory antibiotic) therapy and is now undergoing follow-up, with a significant improvement 6 months after the initial presentation (glomerular filtration rate (GFR) and cystatin C clearance rate of 165 ml/min/1.73m^2^ and 106 ml/min/1.73m^2^, respectively). No signs of bacterial infection were detectable.

**Conclusion:**

This variant of IgA PIGN must be distinguished from other clinical entities, especially IgA nephropathy (mesangial IgA deposits) and postinfectious glomerulonephritis (C3, IgG and occasional IgM capillary loop deposits with or without mesangial distribution), since patients with IgA PIGN may require steroid treatment in addition to antibiotic therapy. Differential diagnosis should also include C_3_ glomerulopathy.

IgA PIGN is a recently identified disease entity that generally manifests in adult patients with both IgA and C3 mesangial and glomerular capillary wall deposits. We present a biopsy-proven case of IgA PIGN that manifested in a patient at an exceptionally young age and that has had a good clinical outcome. To the best of our knowledge, this is the youngest IgA PIGN patient reported thus far.

## Background

IgA-dominant PIGN is a newly recognized variant of PIGN. It is characterized by proliferative glomerulonephritis seen in light microscopy (LM) with dominant or codominant mesangial and glomerular capillary wall deposits of IgA detected by immunofluorescence (IF) in combination with hump-like deposits obtained by electron microscopy (EM) [1].

The Arkana laboratory database gathered more than 80,000 kidney biopsies and identified about 160 cases of IgA PIGN. Patients were all adults, and the majority were over 40 years of age. About 50% had a comorbidity of diabetes mellitus. Many patients were tested positive for methicillin-resistant *Staphylococcus aureus* (MRSA) or methicillin-susceptible *Staphylococcus aureus* (MSSA), and in some of these patients ANCA testing was positive [2, 3]. IF has identified IgA as a dominant or a co-dominant antibody, with a higher potency detected for C3 than IgA. Subepithelial humps and focal necrotizing glomerulonephritis were present in some of the cases [2].

Typical IgA-dominant PIGN has been described in only three children below 18 years of age with a lowest age of 12-years [4, 5]. IgA PIGN typically presents with acute renal failure and severe proteinuria in the majority of cases. Hypocomplementemia is common and can be detected in two-thirds of patients [6].

Conditions predisposing a patient to the develop IgA-dominant PIGN include old age, diabetes, and *Staphylococcus aureus* infections, furthermore cancer, drug and alcohol abuse [6]. IgA PIGN has been linked to staphylococcal infections but also to infections with other pathogens including *E. coli*, *Enterococcus* species and HIV. This type of glomerulonephritis therefore appears to be postinfectious [6–10]. Pharyngitis has been superseded by skin, urinary or pulmonary infections [6, 11–15].

IgA PIGN variants should be differentiated from IgA nephropathy. Features supporting the diagnosis of IgA PIGN over IgA nephropathy include manifestation at an older age, acute kidney failure, a documented staphylococcal infection and low serum complement (C3). Histologic features of IgA PIGN comprise endocapillary hypercellularity, a strong infiltration of neutrophils, a marked deposition of C3 compared to IgA, and characteristic subepithelial humps visible on electron microscopy [1, 2].

The diagnosis of IgA-dominant PIGN is not always easy and the awareness of pediatric IgA-dominant PIGN should improve. This is of importance as it affects the choice of treatment modalities. However, there is no consensus on the management of IgA PIGN. The early use of antibiotics is highly recommended to effectively treat the underlying bacterial infection [16]. However, IgA-dominant PIGN has also been reported without identification of a causal infection [17]. In the presence of progressive glomerulonephritis steroid therapy should be initiated [4, 16]. Despite these recommendations, complete renal recovery was only observed in less than 20% of adult patients [6].

## Case presentation

A 3 year and 2-month-old girl presented to the emergency department with a rash on her legs for 1 day and haematuria for 4 days. There was no accompanying fever. She was started on a course of oral cefaclor for presumed urinary tract infection 2 days ago. Urine culture showed no bacterial growth. Eight weeks earlier, the girl had a purulent nail infection and was treated conservatively. One year earlier, the girl had severe streptococcal tonsillitis. The throat swab at that time was positive for streptococci. Her symptoms improved after 10 days of antibiotic therapy. There was no known kidney disease in the family.

*Physical examination* revealed a hypertensive patient with a rash on her lower extremities but no peri-articular swelling. The rash disappeared on day 4 after admission. Pedal and eyelid oedema were present. No ear, nose or throat (ENT) pathologies, enlarged lymph nodes or ascites were observed.

*Clinical examination* demonstrated Glasgow Coma Scale (GCS) score 15, pulse 101 beats/min, capillary refill time 1 second, blood pressure 104/63 mmHg (99.P), respiration rate of 24 breaths/min, oxygen saturation (SpO_2_) 96%, temperature 36.8 °C, weight 16 kg (75.P), and height 96 cm (39.P).

*Investigations showed negativity for* myeloperoxidase antibodies, lactoferrin antibodies, proteinase 3 antibodies, bactericidal/permeability-increasing protein antibodies, cathepsin G antibodies, antinuclear antibodies and antineutrophil cytoplasmic antibodies and elevated levels of urea, uric acid, creatinine, perinuclear anti-neutrophil cytoplasmic antibodies, elastase antibodies, IgG and anti-streptolysin. The level of C3 was low (0.260 g/l). The results are shown in the supplementary table (Table 1).

**Table 1:** Laboratory results at hospital admission

	Value	Range	Unit
Leukocyte	10.6	5.4–13.8	G/L
Erythrocyte	4.4	3.85–5.15	T/L
Haemoglobin	109	107–139	g/L
Haematocrit	0.33	0.33–0.42	L/L
Platelet	360	200–460	G/L
Neutrophils	73	25–68	%
Lymphocytes	18	28–59	%
Eosinophils	0	0.5–5.0	%
Monocytes	8	1.5–9.0	%
Basophils	0	0–1.50	%
Blood sodium	136	134–143	mmol/L
Blood potassium	4.3	3.3–4.6	mmol/L
Blood magnesium	1.1	0.62–0.95	mmol/L
Blood calcium	2.37	2.20–2.70	mmol/L
Blood phosphorus	2.4	1.0–1.95	mmol/L
Blood chloride	103	96–109	mmol/L
Blood glucose	103	60–100	mg/dl
Protein	71	57–80	g/L
Albumin	28	37–51	g/L
CRP	3.8	< 5	mg/L
PT	100	82–121	%
INR	0.91	0.85–1.15	Ratio
aPTT	25	23–38	sec
Fibrinogen	2.8	1.8–5	g/L
LDH	297	105–338	U/L
Bilirubin (total)	0.25	0.2–1	mg/dl
c-ANCAs	negative	< 10	
p-ANCAs	160	< 10	
Antistaphylolysin	negative	negative	IU/ml
ASO	532	< 200	IkU/L
Serum IgA	1.6	0.3–1.9	g/l
Serum IgG	15	5.4–13.4	g/L
Serum IgM	0.85	0.52–1.9	g/l
Complement C3	0.260	0.8–1.5	g/l
Complement C4	0.220	0.1–0.4	g/L
Blood urea nitrogen	49,5	5–25	mg/dl
Serum uric acid	8	1.8–5.0	mg/dl
Serum creatinine	0.82	0.26–0.77	mg/dl

*CRP* C-reactive protein, *PT* Prothrombin time, *INR* International normalized ratio, *aPTT* Activated partial thromboplastin time, *LDH* Lactate dehydrogenase, *c-ANCAs* Antineutrophil cytoplasmic antibodies, *p-ANCAs* Perinuclear antineutrophil cytoplasmic antibodies, *ASO* Antistreptolysin

*Urinalysis:* Dipstick tests showed 3+ proteinuria, haematuria, and leukocyturia. The results were negative for nitrite and showed a normal urine pH. A 24-h urine test showed *normal* creatinine levels and elevated *levels* of protein(2.8 g/l), protein/g creatinine(7955 mg/g). See (Table 2).

**Table 2:** 24-h Urine test results

	Value	Range	Unit
Urine volume	730 ml		ml/d
Creatinine	35.2		mg/dl
Protein	2.8	< 0.05	g/L
Protein/g creatinine	7955	< 70	mg/g
IgG/g creatinine	233	< 9	mg/g
A2 macroglobulin	19	< 5	mg/L
IgG	82	< 7.8	mg/L
Albumin	1920	0–20	mg/L
A1 microglobulin/g creatinine	28	< 14	mg/g
Albumin/creatinine	2500	< 30	mg/g

**Fig. 1:**
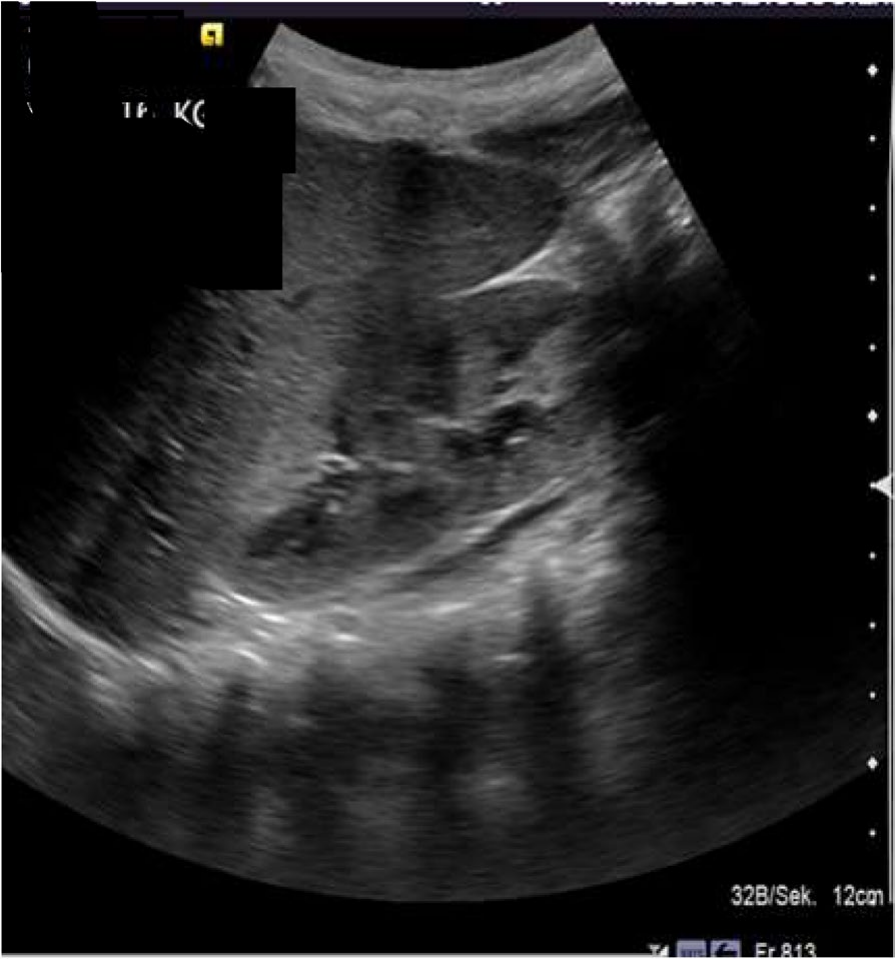
Renal ultrasound showing an enlarged hyperechogenic kidney. Otherwise, this was an age-appropriate abdominal sonogram

Renal ultrasound showed bilateral enlarged hyperechogenic kidneys. The volumes were 64 cm^3^ for the right kidney and 70 cm^3^ for the left kidney. There was no evidence of any urinary transport disorder. Otherwise, an age- appropriate abdominal sonogram was documented (Fig. 1).

*Echocardiography:* There was no left ventricular hypertrophy (LVH) or pericardial effusion. The tricuspid aortic valve and coronary outlets were normal. Physiologic regurgitation was observed in the pulmonary and tricuspid valves. There was no aortic or mitral insufficiency.

**Fig. 2:**
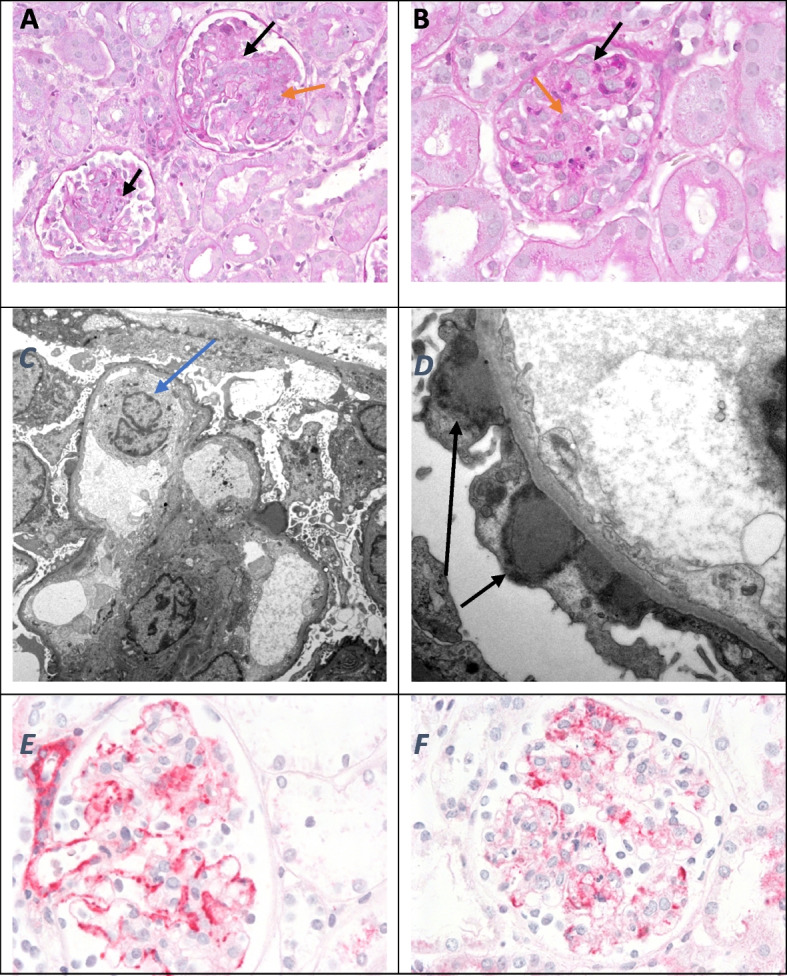
**A** and **B** Light microscopy showed moderate expansion of the mesangial matrix with mesangial hypercellularity and segmental endocapillary hypercellularity (black arrows) with intracapillary neutrophils (orange arrows) (PAS stain). Original magnification × 400. **C** and **D** Electron microscopy showed proliferation of endothelial and mesangial cells and neutrophils (blue arrow) in the lumen. There are several hump-shaped subepithelial electron-dense deposits (black arrows). Original magnification × 5.000 for C and × 8.000 for **D**. **E** and **F** Immunohistology showed dominant staining for IgA and segmental mesangial hypercellularity. (APAAP stain). Original magnification × 400

*Renal biopsy* was performed. *Light microscopy* showed 47 glomeruli, out of which seven showed segmental basal membrane rupture with leakage of necrotic fibrinoid material into the extra-capillary space and adjacent alternating strong extra-capillary proliferation. The remaining glomeruli showed significantly increased mesangial and endocapillary hypercellularity, with focal infiltration of the intra-capillary space by neutrophilic granulocytes and monocytes. The peripheral basal membranes were typical, with podocytes with flat cytoplasm. The afferent arterioles were not affected, and Congo red staining was negative (Fig. 2A and B).

*Electron microscopy* showed glomerular parts with typically structured basement membranes. An increase in mesangial cellularity was observed, along with electron-dense subepithelial humps and mesangial deposits. Endocapillary hypercellularity and granulocytic and monocytic infiltration were prominent (Fig. 2C and D).

*Immunofluorescence* revealed positive focal segmental mesangial and glomerular basement membrane staining for IgA and complement factor (C1q). The same pattern was observed to a lesser extent for IgG. A positive glomerular basement membrane staining in the form of humps for complement factor C3 was observed. A strong complement factor C3 and Fibrinogen staining was detected in the mesangium. The same pattern was observed to a lesser extent for IgM (Fig. 2E and F).

A causative organism was not identified and based on an initial clinical diagnosis of Henoch-Schoenlein purpura with heavy proteinuria, the girl was initially managed with methylprednisolone 300 mg/m^2^ i.v for three alternate days, followed by prednisolone p.o. 40 mg/m^2^. Renal biopsy findings were then consistent with IgA PIGN, and the patient was treated with 10 days of antibiotic therapy consisting of cefuroxime i.v. for 3 days followed by flucloxacillin p.o. for 7 days due to a presumed untreated staphylococcal skin infection (paronychia) 8 weeks prior. Prednisolone 40 mg/m^2^ p.o. was continued for a total period of 1 week and then weaned over next 6 weeks.

The patient experienced partial renal recovery (decrease in serum creatinine from 0.82 mg/dl to 0.31 mg/dl) and a reduction in nephrotic proteinuria (decline from 7955 mg/g to 782 mg/g creatinine) within 32 days. Her serum C_3_ level completely normalized on day 25 after admission.

At the 6-month follow-up visit, the patient’s creatinine level had improved to 0.25 mg/dL, with a urine protein to creatinine (UPC) ratio of 132 mg/g.

## Discussion and conclusion

IgA PIGN is a rare but increasingly recognized PIGN variant. It has been reported in only 3 children worldwide, the youngest being 12 years old [4, 5].

We here present a 3-year-old child that manifested with nephritic and nephrotic syndrome in the setting of a recent paronychia. She was treated with steroids and antibiotics which was associated with an improvement in kidney function and level of proteinuria. This report raises awareness regarding IgA-dominant PIGN, especially in children. Factors that support this rare diagnosis in this age group include hypocomplementaemia, recent suspected staphylococcal infections and characteristic histological findings in renal biopsy specimens.

IgA dominant PIGN is a recently recognized entity with rising incidence that has to be distinguished from the classical diagnosis of IgA nephropathy on one hand and post-streptococcal PIGN on the other. IgA dominant PIGN and IgA nephropathy share clinical and histological similarities that can mislead in diagnosis, especially if the GN remains undiagnosed for a long time. Satoskar et al. summarized clinical and histological factors that are useful in distinguishing these two disease entities [18, 19]. Leading clinical features that favour IgA PIGN over IgA nephropathy are manifestation at an older age, acute kidney failure, a documented staphylococcal infection and low serum complement (C3). Typical histologic features of IgA PIGN include diffuse endocapillary hypercellularity, marked neutrophil infiltration, enhanced deposition of C3 compared to IgA, and characteristic subepithelial humps visible on electron microscopy [4, 20–22]. If several of the above factors are present, IgA dominant PIGN is a likely diagnosis.

Furthermore, IgA nephropathy and IgA PIGN differ in mesangial staining for IgA1 lambda and kappa isoform ratio, which shows a dominance of IgA1 lambda in IgA nephropathy and of IgA1 kappa in IgA PIGN [4, 17, 23].

Unlike classical post-streptococcaI PIGN, which is characterized by C3 deposition, IgA PIGN has a typical IgA deposition [6]. The fact that infection with *Staphylococcus* spp. can cause either IgA PIGN or acute classic PIGN suggests the existence of additional factors causing renal disease. The type of infection does not completely explain the observed differences in antibodies production. Genetic susceptibility may also play a role [3, 19]. To date, the pathophysiology of IgA dominant PIGN remains unknown to a large extend and some might consider it to be a variation of IgA nephropathy.

Renal biopsy confirmed the diagnosis of IgA PIGN in our patient. Unlike the findings in the study by Nasr et al. [6], in which the subepithelial deposits in cases of IgA PIGN were small and sparse, our findings are therefore more consistent with earlier observations by Haas et al. [4, 24], in that subepithelial deposits were large, numerous, and hump shaped.

To date, there has been no systematic assessment of treatment choices or their effectiveness in the treatment of IgA-dominant PIGN, including steroids [6, 25]. The clinical response is usually favourable when the underlying infection is treated, but in some cases, steroid treatment has been added [7–9, 20]. Clinical criteria for the use of steroids are not well defined. It has been suggested that steroid therapy should be considered in patients who do not respond to antibiotics alone [26].

In our report, the patient was followed for a short period (< 1 year) and experienced partial recovery from renal failure and persistent proteinuria. Long-term follow-up is needed to assess the progression of the disease. Nasr et al. noted that only 16% of adult IgA-dominant PIGN patients experienced full recovery of renal function (≤1.2 mg/dl), whereas 43% had persistent renal dysfunction, and 41% progressed to end-stage renal disease (ESRD) [6].

In summary, the prognosis of IgA-dominant PIGN is less favourable than that of typical acute post-infectious glomerulonephritis [4, 12–14, 20, 22, 27], in the majority of cases, 70 to 80% start with acute kidney injury, proteinuria, haematuria, and hypocomplimentaemia [4, 6, 12–14, 20, 22, 27]. Despite the fact that IgA PIGN seems to be a disease without recurrences and exacerbations that characterize primary IgA nephropathy, only less than 20% of adult patients achieve a complete renal recovery. Data on paediatric patients is scarce and it remains to be observed and discussed in future publications whether the renal prognosis in paediatric age group is more favourable than in adults and elderly.

## Data Availability

The datasets used and/or analysed during the current study are available from the corresponding author on reasonable request.

## Abbreviations

IgA: Immunoglobulin A
IgA PIGN: immunoglobulin A-dominant postinfectious glomerulonephritis
PIGN: post-infectious glomerulonephritis
LM: light microscopy
IF: immunofluorescence
EM: electron microscopy
BPI: bactericidal/permeability-increasing protein
UPC: urine protein to creatinine
